# A Novel Fault Diagnosis Method of High-Speed Train Based on Few-Shot Learning

**DOI:** 10.3390/e26050428

**Published:** 2024-05-16

**Authors:** Yunpu Wu, Jianhua Chen, Xia Lei, Weidong Jin

**Affiliations:** 1 School of Electrical and Electronic Information, Xihua University, Chengdu 610039, China; chenjianhua@stu.xhu.edu.cn (J.C.); snow_lei246@mail.xhu.edu.cn (X.L.); 2 School of Electrical Engineering, Southwest Jiaotong University, Chengdu 611756, China; wdjin@home.swjtu.edu.cn

**Keywords:** high-speed train, fault detection, few-shot learning, meta learning

## Abstract

Ensuring the safe and stable operation of high-speed trains necessitates real-time monitoring and diagnostics of their suspension systems. While machine learning technology is widely employed for industrial equipment fault diagnosis, its effective application relies on the availability of a large dataset with annotated fault data for model training. However, in practice, the availability of informational data samples is often insufficient, with most of them being unlabeled. The challenge arises when traditional machine learning methods encounter a scarcity of training data, leading to overfitting due to limited information. To address this issue, this paper proposes a novel few-shot learning method for high-speed train fault diagnosis, incorporating sensor-perturbation injection and meta-confidence learning to improve detection accuracy. Experimental results demonstrate the superior performance of the proposed method, which introduces perturbations, compared to existing methods. The impact of perturbation effects and class numbers on fault detection is analyzed, confirming the effectiveness of our learning strategy.

## 1. Introduction

High-speed railways have become a crucial mode of transportation in modern society, offering advantages such as time efficiency and convenience for passengers. The stability and safety of high-speed trains are paramount, considering their high-speed nature. The suspension system plays a pivotal role in maintaining this stability, consisting of key components like coil springs, air springs, and hydraulic dampers. Any malfunction in these components poses a potential threat to the safe operation of the train and the wellbeing of passengers. Therefore, monitoring the health of critical components in the suspension system of high-speed trains holds significant importance [1]. Ensuring the continuous and accurate diagnosis of faults in these components is essential for maintaining the safety and reliability of high-speed train operations.

Various methodologies exist for diagnosing faults in high-speed train components, encompassing expert knowledge-based, model-based, and data-driven approaches [2,3]. Among these, deep learning, a subset of data-driven techniques, has gained prominence for its capabilities in extracting intricate features from data [4,5]. Noteworthy architectures such as stacked autoencoders, deep belief networks, and convolutional neural networks are commonly employed in fault diagnosis. These models serve either as feature extractors or as end-to-end structures, showcasing advantages in adaptive feature extraction and comprehensive fault analysis [6,7]. Recent studies have explored fault diagnosis methods for high-speed train components, including traction systems [5], running gears [8], bogies [9], and yaw dampers [10]. Additionally, hybrid models integrating physical and data-driven approaches have been proposed for fault detection in axle bearings [11]. Despite their successes, these approaches face challenges, particularly in the need for substantial labeled data. The limited availability of fault samples poses a significant constraint on the practical application of deep learning models for fault diagnosis in high-speed train components. Overcoming this limitation and effectively handling the scarcity of labeled data remain critical aspects for further advancing the field of high-speed train fault diagnosis.

Few-shot learning emerges as a promising solution for addressing the challenges of data scarcity in fault diagnosis, especially in scenarios where obtaining abundant labeled data is impractical. This approach involves training models to recognize new fault classes with minimal labeled examples, making it adaptable to situations with limited data availability. Few-shot learning’s effectiveness extends beyond traditional machine learning limitations, finding applications in various domains where data is scarce. In the context of fault diagnosis, few-shot learning becomes particularly relevant by requiring only a small number of labeled samples for each fault class. This adaptability is crucial in overcoming challenges associated with acquiring extensive labeled data, a common constraint in fault diagnosis applications. Few-shot learning’s ability to generalize from limited examples makes it well-suited for the dynamic and diverse nature of fault patterns in high-speed train components. The efficacy of few-shot learning in fault diagnosis is demonstrated through diverse strategies, including data augmentation-based methods, meta-learning approaches, distance metric-based techniques, and migration learning-based methods. These methodologies within the few-shot learning framework contribute to enhancing fault diagnosis accuracy, especially in the presence of limited labeled data. The efficacy of few-shot learning in fault diagnosis is demonstrated through diverse strategies. Snell et al. [12] introduce a simple yet effective approach for few-shot learning by learning prototype representations of each class in a metric space. Finn et al. [13] propose Model-Agnostic Meta-Learning method for few-shot learning, which is compatible with any model trained with gradient descent and applicable to a variety of different learning problems. Ren et al. [14] extend Prototypical Networks to incorporate unlabeled examples within each episode, demonstrating improved predictions akin to semi-supervised algorithms. Liu et al. [15] propose Transductive Propagation Network (TPN) for transductive inference in few-shot learning, addressing the low-data problem by learning to propagate labels from labeled instances to unlabeled test instances. Notably, transductive inference is a flavor of few-shot learning that has gained attention for its ability to leverage unlabeled data for better generalization. This characteristic is particularly advantageous in fault diagnosis, where labeled data is often limited, and the inclusion of unlabeled data can significantly improve model performance. As the field progresses, the application of few-shot learning principles is expected to play a pivotal role in advancing fault diagnosis capabilities, providing effective solutions for real-world scenarios characterized by data scarcity.

Recent advancements in few-shot learning for fault diagnosis have yielded diverse methodologies tailored to mitigate the challenges of limited data availability. These approaches encompass various strategies, including meta-learning frameworks [16,17], which address data scarcity by leveraging innovative decomposition methods and model-agnostic meta-learning strategies integrated with specialized frameworks. Additionally, Ref. [18] introduces a multimodal few-shot learning framework adept at handling unbalanced data in industrial bearing fault diagnosis, while Cen et al. [19] propose an anomaly detection model for industrial motors that utilizes reinforcement and ensemble learning under few-shot feature conditions. Moreover, methods like meta-transfer learning [20], customized meta-learning frameworks [21], and efficient two-stage learning frameworks [22] offer innovative solutions to address domain-shift challenges and enhance feature invariance to data shifts, ultimately improving fault diagnosis performance. These studies collectively underscore the versatility and efficacy of few-shot learning techniques in fault diagnosis applications.

In this context, although previous research has explored the application of few-shot learning in fault diagnosis, it has largely overlooked the uncertainty of samples from unknown distributions in fault diagnosis tasks. This uncertainty can lead to misdiagnosis of faults and result in serious consequences. Additionally, the lack of targeted regularization methods, such as signal-specific data augmentation techniques, to address the overfitting problem in few-shot learning for fault diagnosis tasks has also constrained the performance of models. This paper introduces a novel few-shot learning approach, denoted as Sensor-Perturbation Injection and Meta-Confidence Learning (SPI-MCL), designed for diagnosing high-speed train faults. The methodology involves mapping input data from various tasks to a shared feature space using one-dimensional convolutional neural networks. Each query sample in each class is then assigned a distinct confidence score based on a distance metric formula in this feature space. Subsequently, weighted averages of confidence scores are computed to update class prototypes, thereby enhancing fault classification. Given the non-overlapping nature of training and test classes, the classification of unknown samples in the test set may be unreliable. To mitigate this concern, we introduce sensor-wise data perturbation and model perturbations during the meta-learning process to bolster the reliability of output confidence scores. The designed sensor-wise perturbation can generate different perturbation modes for each sensor and accommodate multichannel scenarios in high-speed train fault diagnosis, where monitoring signals from different sensors exhibit varying distributions and characteristics. This injection of randomness facilitates better learning of confidence measures, consequently improving fault classification accuracy. Our key contributions encompass:

(1) Proposing a novel approach for fault diagnosis based on meta confidence learning.
(2) Enhancing fault detection performance through the injection of sensor-wise perturbations.
(3) Validating the effectiveness of the proposed method on a high-speed train fault diagnosis dataset.

## 2. Method

This section provides a detailed description of the proposed SPI-MCL method, which is designed for high-speed train fault diagnosis. The methodology employs a neural network model, and involves two main techniques: meta-confidence learning (in Section 2.2) for learning confidence scores and updating prototypes, and sensor-wise perturbations (in Section 2.3) to enhance the model’s capability for extracting features from nonlinear signals. The overall framework of the proposed method is presented in Section 2.4.

### 2.1. Few-Shot Classification and Prototype-Based Method

The detection task for fault types with limited labeled data can be seen as a few-shot classification problem, a scenario frequently encountered in fault diagnosis applications. In the realm of few-shot classification, particularly relevant to fault detection, the task is often termed *K*-way *N*-shot classification. Here, *K* represents the number of fault classes, and *N* denotes the limited number of labeled samples available per fault class for training. In practical terms, this can be likened to the scenario where each fault class has a sparse set of exemplar samples for model learning.

The setup involves a support set (S) and a query set (Q). The support set includes *K* classes, each with *N* samples, denoted as $S={\left\{\left(x_{i},y_{i}\right)\right\}}_{i=1}^{K\times N}$. The query set, used for evaluating the model’s performance, also comprises *K* classes, but with *M* samples per class, represented as $Q={\left\{\left(x_{i},y_{i}\right)\right\}}_{i=1}^{K\times M}$. In the context of fault detection, this aligns with the practical challenge of learning from a small number of labeled samples for each fault type in the training set and subsequently validating the model on a similarly limited dataset.

A notable approach in few-shot learning, particularly relevant to fault diagnosis, is the Prototype-based method [12]. This method addresses the challenge by learning a prototype $P_{k}=\frac{1}{|S_{k}|}\sum f_{\theta }\left(x\right)$ for each fault class, where $S_{k}$ represents the set of labeled samples with *k* class, and θ represents learnable parameters. In the fault diagnosis context, the prototype can be conceptualized as a representative reference or average feature set of the support samples within a given fault class. The classification of samples in the query set is then determined based on the distance metric between the prototype and the query sample. This methodology is well-suited for fault detection scenarios where learning from a limited number of labeled samples is a common challenge, enabling effective generalization and discrimination among fault classes in the presence of sparse training data.

### 2.2. Meta-Confidence Learning with Transductive Inference

The prototype-based method has shown its effectiveness in many related tasks. However, the original prototype-based method does not consider the uncertainties of prediction on an unseen task, which may cause serious consequences, especially in fault diagnosis tasks. In fault diagnosis scenarios, where faults may exhibit similar characteristics leading to confusion or where fault features vary, addressing prediction uncertainties becomes crucial for reliable diagnosis. Meta-confidence learning [23] provides a feasible solution with transductive inference. The method leverages the unlabeled examples for refining prototypes by updating them according to the confidence score [14]. The concept behind meta-learning is that the information gain obtained from learned instances should prove valuable for analyzing future instances.

The method is described as follows: First, the initial prototype for each class k=1…K is computed as $P_{k}^{\left(0\right)}=\frac{1}{|S_{k}|}\sum_{x\in S_{k}}f_{\theta }\left(x\right)$. Subsequently, for each step t=1…T, and for each query example $\tilde{x}\in Q_{x}$, the confidence score $q_{k}^{\left(t-1\right)}\left(X\right)$ is determined, representing the probability of it belonging to each class *k*, according to the equation:

(1) $$q_{k}^{\left(t-1\right)}\left(X\right)=\frac{\exp(-d\left(f_{\theta }\left(X\right),P_{k}^{\left(t-1\right)}\right))}{\sum_{k^{\prime }=1}^{k}\exp\left(-d\left(f_{\theta }\left(\tilde{x}\right),P_{k}^{\left(t-1\right)}\right)\right)}$$

where *d* denotes the Euclidean distance and $P^{\left(t-1\right)}$ represents the prototype updated up to step t−1.

The prototypes of class *k* are then updated based on the confidence scores (or soft labels) $q_{k}^{\left(t-1\right)}\left(X\right)$ for all X∈Qx, given by the following:

(2) $$P_{k}^{\left(t\right)}\;=\frac{\sum_{x\in S_{k}}1\cdot f_{\theta }\left(x\right)+\sum_{X\in Q_{x}}q_{k}^{\left(t-1\right)}\left(\tilde{x}\right)\cdot f_{\theta }\left(\tilde{x}\right)}{\sum_{x\in Sk}1+\sum \tilde{x}\in Q_{x}q_{k}^{\left(t-1\right)}\left(\tilde{x}\right)}$$

which represents the weighted average. It is noted that the confidence of the support examples is invariably 1, given their observed class labels. The process is iteratively repeated until t=1…T. The confidence scores reflect the model’s certainty in its predictions, crucial for distinguishing between similar faults or handling variations in fault characteristics.

Specifically, the distance metric $d_{\phi }$ is meta-learned, where it is defined as the Euclidean distance with normalization and instance-wise or pair-wise metric scaling, denoted as $g_{\phi }^{I}$ and $g_{\phi }^{P}$, respectively:

(3) $$d_{\phi }^{I}\left(a_{1},\;a_{2}\right)={∥\frac{a_{1}/\left|\right|a_{1}{∥}_{2}}{g_{\phi }^{I}\left(a_{1}\right)}-\frac{a_{2}/\left|\right|a_{2}{∥}_{2}}{g_{\phi }^{I}\left(a_{2}\right)}∥}_{2}^{2}$$

(4) $$d_{\phi }^{P}\left(a_{1},\;a_{2}\right)={∥\frac{a_{1}/{∥a_{1}∥}_{2}}{g_{\phi }^{P}\left(a_{1},a_{2}\right)}-\frac{a_{2}/{∥a_{2}∥}_{2}}{g_{\phi }^{P}\left(a_{1},a_{2}\right)}∥}_{2}^{2}$$

for all $a_{1},a_{2}\in R^{l}$, where $a_{1},a_{2}$ are the *l*-dimensional feature vector generated by the network model from two data samples. The normalization ensures that the confidence is primarily determined by metric scaling. To obtain the optimal scaling function $g_{\phi }\in g_{\phi }^{I},\;g_{\phi }^{P}$ for transduction, the query likelihoods after *T* transduction steps are computed first, followed by the optimization of ϕ, the parameter of the scaling function $g_{\phi }$, through minimizing the instance-wise loss for $d_{\phi }\in d_{\phi }^{I},\;d_{\phi }^{P}$:

(5) $$\begin{array}{cc} L_{I}^{\tau }\left(\theta ,\;\phi \right) & =\;\frac{1}{\left|Q\right|}\sum_{(X,\tilde{y})\in Q}-\log p\left(\tilde{y}|X,\;S;\theta ,\;\phi \right)\\ \; & =\;\frac{1}{\left|Q\right|}\sum_{(\tilde{x},\tilde{y})\in Q}d_{\phi }\left(f_{\theta }\left(X\right),\;P_{c}^{\left(T\right)}\right)+\sum_{c=1}^{C}\exp\left(-d_{\phi }\left(f_{\theta }\left(X\right),\;P_{c}^{\left(T\right)}\right)\right) \end{array}$$

Regarding $g_{\phi }$, a convolutional neural network with fully-connected layers can be utilized, which takes either the feature map of an instance or the concatenated feature map of a pair of instances as input. The meta-learning of distance metrics allows the model to adapt to variations in fault characteristics and similarities, ensuring reliable diagnosis in diverse fault scenarios.

In few-shot classification, to enhance the robustness and generalization capability of the model to the samples, a feasible approach is to inject perturbations into the samples. By introducing various types of perturbations, the model can better adapt to different data distributions and features during the training process, thus improving its ability to recognize unseen samples. Additionally, perturbation injection helps prevent the model from overfitting to the training data, facilitating the model to better capture the underlying features among samples in few-shot learning tasks. In [23], both data perturbation and model perturbation are utilized to output more reliable and consistent confidence.

In this section, we proposed sensor-wise perturbations into the fault detection process. By adding sensor-wise perturbations to the monitoring data, the model can better adapt to different data distributions and features during the training process, thus improving its ability to recognize unseen samples. The introduction of sensor-wise perturbations during training induces controlled entropy in the model’s decision boundaries, allowing it to learn more nuanced and robust representations of the input data. This approach enables it to effectively capture intricate patterns in the data for fault diagnosis tasks.

### 2.3. Sensor-Wise Perturbation

**Figure 1:**
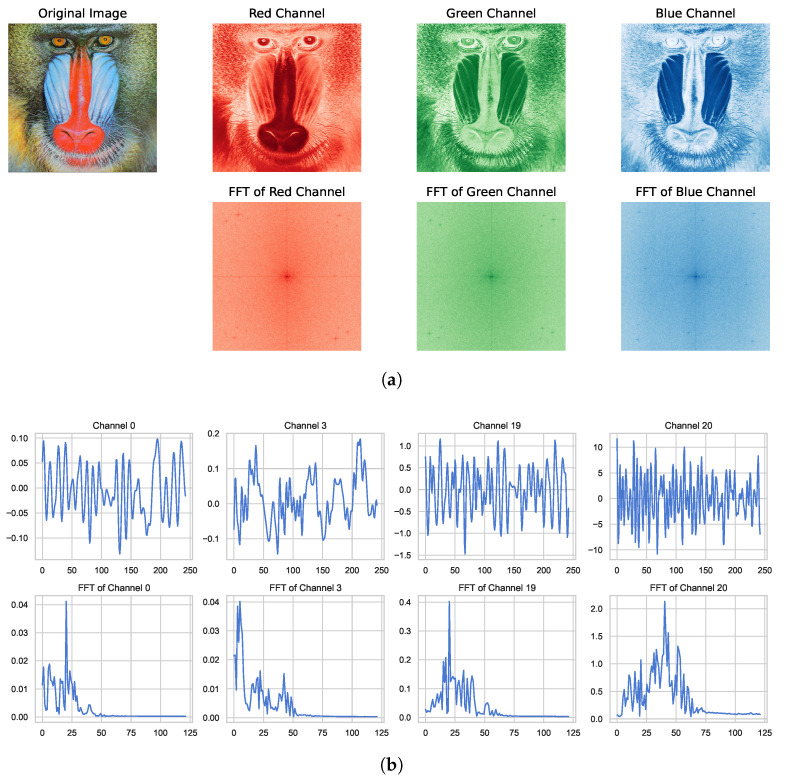
Comparison of images and vibration signals in different channels. (a) RGB channels of the image and their 2D FFT; (b) Multiple channels of vibration sensors and their FFT.

The motivation behind sensor-wise perturbation is based on the following considerations. Unlike image data and other similar formats, multi-sensor monitoring signals possess their own characteristics. For typical natural image data, the three color channels commonly exhibit the same range and similar distribution. Therefore, perturbations and data augmentation techniques for image data typically treat the entire image data without distinguishing between channels. However, for sensor monitoring signals, data from different sensors usually have different ranges and distributions, especially for coupled mechanical systems. For instance, low-frequency vibrations at one monitoring point may induce high-frequency responses at another monitoring point, resulting in inconsistent distributions of key modal identification features across different monitoring channels. Here, Figure 1 demonstrates the similarities and differences between different channels of image data and the vibration monitoring data addressed in this paper. The proposed sensor-wise perturbation in this section specifically addresses the perturbation techniques related to the characteristics of sensor monitoring signals, aiming to enhance the distribution of data in scenarios of limited sample learning.

The specific steps of sensor-wise perturbation can be described as follows: Firstly, for a monitoring signal *x* with *C* channels, perform fast Fourier transform (FFT) on all channels to obtain *X*. Then, compute the sensor-wise perturbation threshold based on the amplitude spectrum A=|X|, where $\theta_{c}=\max\left(A_{c}\right)*\alpha_{t}$, c=1,2,⋯,C, and $\alpha_{t}$ is a scale factor for tuning the perturbation threshold. Subsequently, apply random perturbation to the parts of the amplitude spectrum that exceed the threshold, where $\delta =\epsilon *\mathrm{std}\left(A\right)*\alpha_{n}$, $\epsilon ∼N(0,\sigma^{2})$, $\mathrm{std}(A_{c})$ denotes the standard deviation of the amplitude spectrum for channel *c*, and $\alpha_{n}$ is a scale factor for tuning the noise level. Regarding the perturbations mentioned above, parameter $\alpha_{t}$ controls how many frequency components will be perturbed, while parameter $\alpha_{n}$ controls the intensity of the perturbation. Finally, perform inverse transform on the perturbed spectrum to obtain the perturbed signal, and superimpose Gaussian white noise $\eta_{P}wn$ to simulate the noise characteristics of real monitoring signals. Equation (6) shows the detailed steps of sensor-wise perturbation.

(6) $$\begin{array}{cccc} A & =|\mathrm{FFT}(x)|\\ \theta_{c} & =\max\left(A_{c}\right)*\alpha_{t}, & c & =1,2,\cdots ,C\\ \delta_{i} & =\epsilon *\mathrm{std}\left(A_{c}\right)*\alpha_{n}*H\left(\delta ,\theta_{c}\right)\; & \epsilon & ∼N\left(0,\sigma^{2}\right),\;H\left(\delta ,\theta_{c}\right)=\left\{\begin{array}{cc} 1 & \mathrm{if}\;A_{c(i)}\geq \theta_{c}\\ 0 & \mathrm{if}\;A_{c(i)}<\theta_{c} \end{array}\right.\\ \hat{x} & =\mathrm{IFFT}\left(A+\delta \right)+\eta_{wn} \end{array}$$

**Figure 2:**
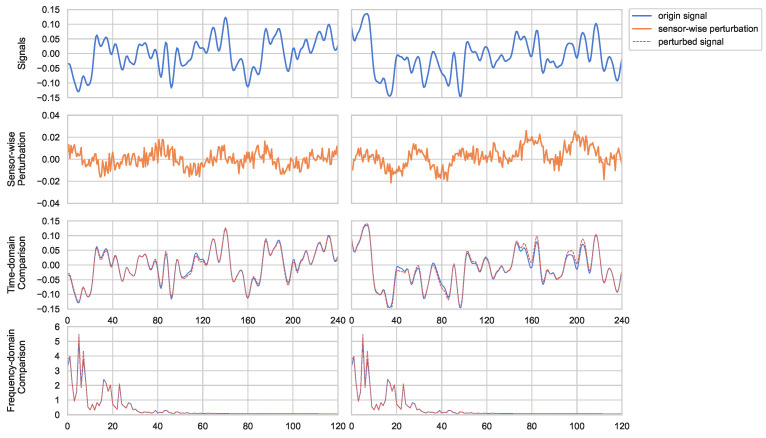
The effect of perturbation on different signals. The blue solid line represents the original signal, the red dashed line represents the signal after perturbation, and the orange solid line represents the injected perturbation.

The advantage of sensor-wise perturbation lies in its ability to introduce variation to vibration-like signals. By perturbing the main frequency components of the signal based on the frequency characteristics of different sensor channels, sensor-wise perturbation ensures that these perturbations are reflected in the time domain while maintaining consistency in the spectral features. Such perturbation enhances the fit of the sample distribution for models trained with limited data, thereby improving the generalization capability of the model. Figure 2 provides an illustrative example of sensor-wise perturbation applied to a vibration signal, which can be seen to introduce variation in the signal while preserving its spectral features. The introduction of sensor-wise perturbations aligns with the intrinsic characteristics of fault diagnosis tasks. By perturbing the data at the sensor level, the model becomes more adept at capturing subtle variations in sensor readings that may indicate fault conditions. Furthermore, the channel-wise nature of the perturbations ensures that the model learns to differentiate between various sensor channels, enhancing its ability to pinpoint the source of anomalies. The introduction of data perturbations enhances the uncertainty in the model’s predictions, enabling it to focus on regions of the feature space with higher information gain and adapt to varying data distributions. This approach aligns with the requirements of fault diagnosis applications, where precise identification of sensor-specific deviations is crucial for accurate diagnosis and maintenance decisions.

### 2.4. Overall Framework

**Figure 3:**
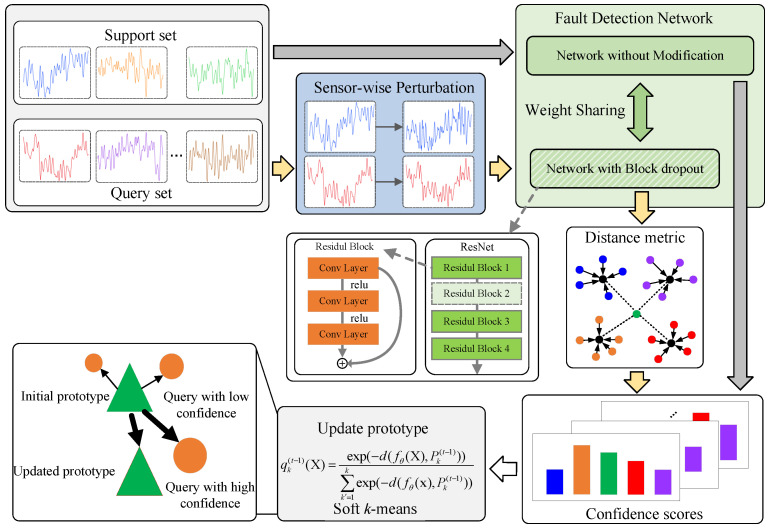
Overll Framework. Sensor-wise perturbations are randomly added to the entire data within each episode to enhance the model’s generalization capability in the face of data uncertainty. The last residual block of the residual network is randomly dropped to capture model uncertainty, representing a form of model perturbation. Meta-learning is employed to adaptively adjust the distance metric based on input data, aiming to enhance the transductive inference performance amid these perturbations.

The overall framework of the proposed method is depicted in Figure 3. The monitoring signal samples are divided into a support set and a query set based on whether they have labels for the components’ conditions (normal or fault) in an episode, which represents a training cycle. The input samples are fed into the model through two pipelines to generate confidence scores. One pipeline involves feeding the original samples into the neural network without any model perturbation, while the other pipeline introduces model perturbation by randomly dropping the last residual block in the residual network and sensor-wise perturbation by adding sensor-wise perturbation to the entire data in the episode. The confidence scores from these two pipelines are then combined as inputs to the soft k-means algorithm for updating prototypes. The initial prototypes for both pipelines are derived by averaging the embeddings of the support set, which are then used to compute confidence scores for each space and class. Then, the prototypes for each space are updated using the ensemble confidence scores obtained from various spaces and queries. This updating process is repeated T times, with each update incorporating an averaged confidence. Finally, inference is performed based on $q_{k}^{\left(t-1\right)}$.

## 3. Experiments

In this section, we present the experimental results of the proposed method on high-speed train fault diagnosis datasets and a public benchmark dataset. The datasets and experimental settings are detailed in Section 3.1. The fault detection performance of the proposed method is presented in Section 3.2, as well as the ablation experiments and comprehensive analysis. The effectiveness of sensor-wise perturbations and meta-confidence learning is demonstrated through the experimental results.

### 3.1. Data Description and Experimental Setting

In this section, aiming to assess the performance of the proposed method, we conduct experiments on the fault diagnosis dataset of high-speed train (HST), which contains monitoring data of the vehicle. Apart from the normal state, the dataset includes 30 classes of failure modes, such as air springs, axle-box springs, and three types of dampers (lateral, yaw, and vertical) on different positions of the suspension system. The failure modes of air springs are often caused by air leakage, the coil springs are prone to breakage, and the failure modes of dampers are often caused by oil leakage or mechanical damage. The actual experimentation involved in studying high-speed train failures is prohibitively expensive and risky. Therefore, to generate monitoring data under various operational conditions, multibody dynamics simulations are conducted to simulate the behavior of the high-speed train [9].

**Figure 4:**
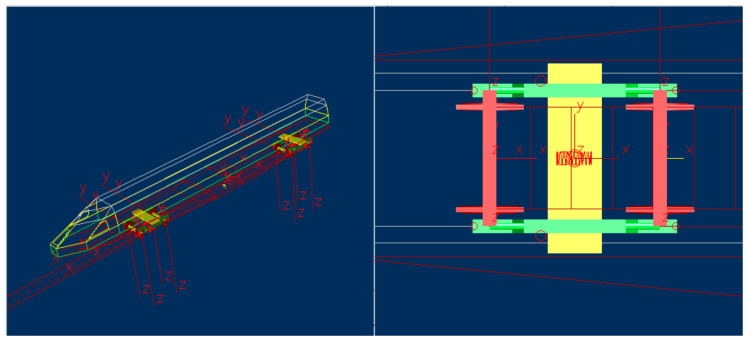
Multibody dynamics simulation model of the high-speed train.

**Figure 5:**
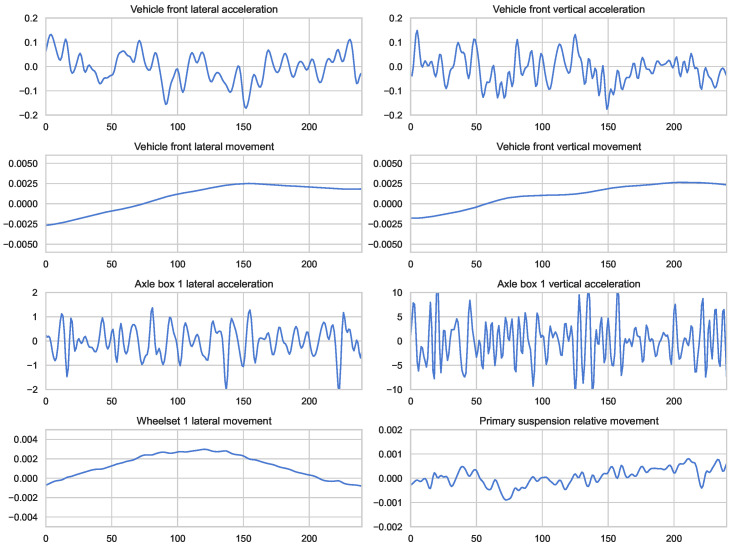
Monitoring signals in the dataset.

The dataset for faults in high-speed train bogies was obtained from simulations conducted on a platform provided by the State Key Laboratory of Traction Power at Southwest Jiaotong University. Aligned with the suspension parameters of the high-speed train CRH380A, dynamic parameters for the simulation were derived from the roller test rig of the railway vehicle, closely mimicking operational conditions. Utilizing the multi-body dynamics analysis software Simpack (version 8.9) (see Figure 4), the simulation platform incorporates geometric and creep nonlinearities, along with nonlinear suspension characteristics. Validation of the simulation was performed through dynamic tests on the roller test rig. During simulation, the vehicle’s dynamic behavior was simulated under a track irregularity spectrum obtained from measurements on the Wuhan-Guangzhou High-Speed Railway. This spectrum approximates real-world conditions, capturing track irregularities and other relevant factors. Monitoring signals were collected using 58 sensors, capturing various motion characteristics. Figure 5 illustrates eight channels of monitoring signals corresponding to normal conditions, encompassing accelerations and displacements of the vehicle’s front section in lateral and vertical directions.

Monitoring data is acquired from sensors installed on the vehicle, capturing accelerations and displacements of the train body, bogie, and wheelset in lateral, longitudinal, and vertical orientations. In total, 58 channels of monitoring data are collected at a sampling frequency of 243 Hz. The classes in the dataset are divided into training and test sets for the experiments, in which the training set includes normal condition, air spring fault, lateral damper fault, yaw damper fault, and the test set includes spring fault and vertical damper fault. Then, sliding windows with a width of 243 points are applied to the monitoring data to obtain samples. The sliding step for both train set and test set are 243 points which means no overlap between the samples. The training and test sets each consist of 500 samples per class. The detailed settings are shown in Table 1.

To ensure experimental comparability, we conducted experiments on the publicly available Case Western Reserve University (CWRU) bearing dataset [24]. This dataset encompasses vibration signals from bearings exhibiting diverse fault types, such as inner race faults, outer race faults, and ball faults. We partitioned the dataset into training and testing sets, with the training set comprising normal states and faults at the fan end (inner race faults, outer race faults, and ball faults), while the testing set includes faults at the drive end. Each class in the training set and testing set has 500 samples. Detailed partitioning information is provided in Table 2.

For the transductive inference, the number of transduction steps for training is set to T=1, and the number of transduction steps for testing is set to T=10. The experiments are conducted with five-way classification for training and five-way for test. The query examples for each class are set to ten for training and testing.

### 3.2. Experimental Results

#### 3.2.1. Fault Detection Performance

**Figure 6:**
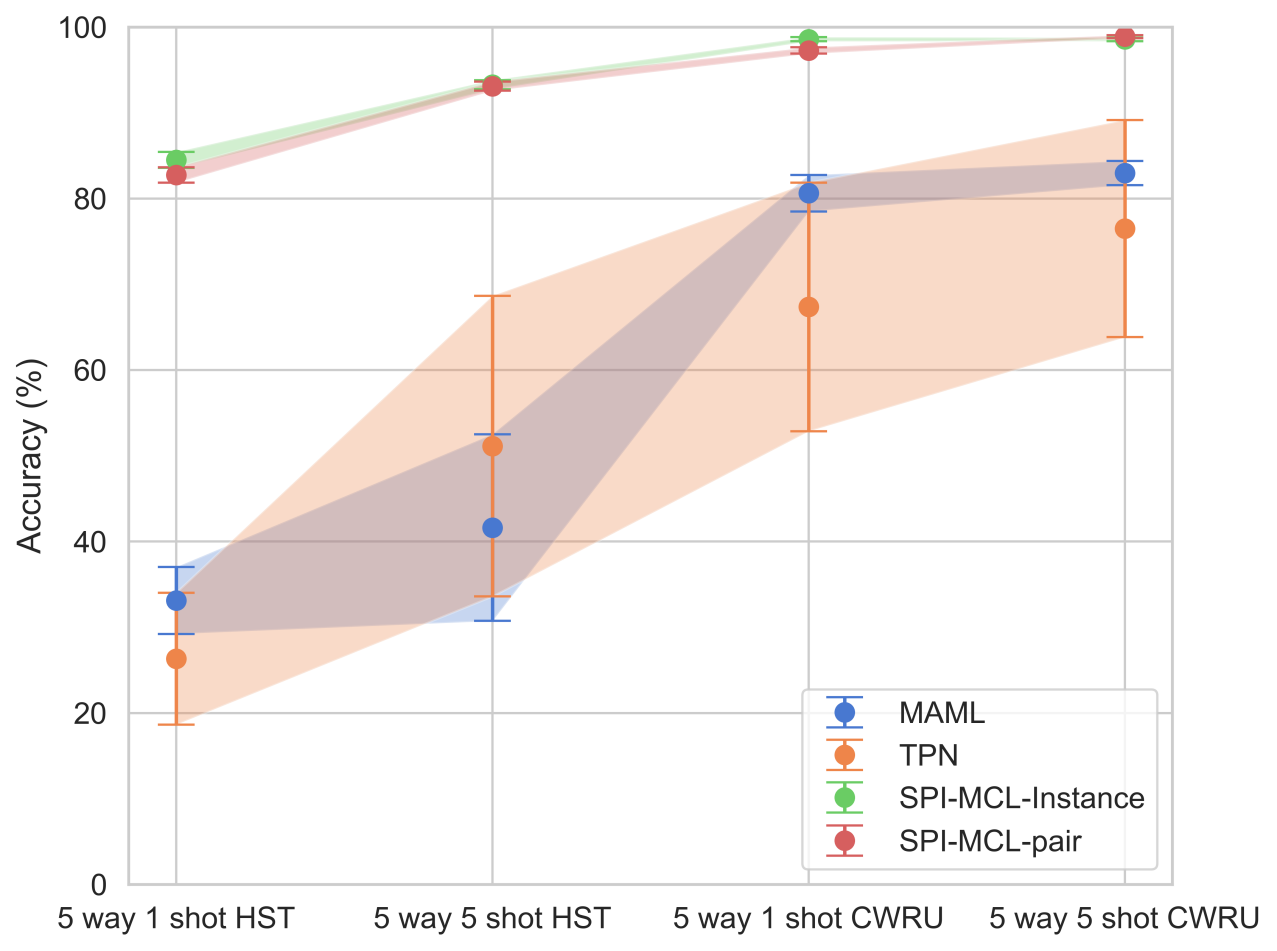
Average detection accuracy of different methods under various settings.

In this section, we conducted comparative experiments on a dataset containing faults in high-speed train suspension systems to evaluate the performance of the proposed SPI-MCL method. These experiments were meticulously designed to compare SPI-MCL with two state-of-the-art few-shot learning methods: Transductive Propagation Network (TPN) [15] and Model-Agnostic Meta-Learning (MAML) [13]. The terms “instance” and “pair” in the table represent instance-wise and pair-wise metric scaling, respectively, as defined in Equations (3) and (4). The backbone network used in the experiments was a ResNet12 network with one dimension convolutional layers and residual blocks, as detailed in Table 3, similar to the architecture used in [15,23,25]. The performance of these methods was evaluated across two distinct datasets: HST→ high-speed train suspension fault dataset and CWRU→ bearing dataset. The experimental settings included five-way classification with one-shot and five-shot scenarios, with the results presented in Table 4 and Figure 6 in which the shaded areas represent standard deviations of the results. The experimental results provide compelling evidence of the superior performance of the SPI-MCL method across all settings. Notably, both SPI-MCL-Instance and SPI-MCL-Pair consistently outperformed TPN and MAML in terms of classification accuracy across different shot numbers. Furthermore, the performance of SPI-MCL exhibited a positive correlation with the number of shots, indicating its capability to effectively leverage limited labeled data for fault diagnosis tasks. These results underscore the robustness and adaptability of the SPI-MCL method, even under conditions of limited labeled data.

#### 3.2.2. Ablation Study of Perturbation

In this section, we explore the impact of different perturbation strategies in the developed scheme. The ablation study, as detailed in Table 5, meticulously dissects the performance of the SPI-MCL method under diverse configurations across two datasets: HST and CWRU. Through a systematic organization of experiments into three distinct groups based on the presence or absence of data and model perturbations, our study offers a nuanced understanding of the algorithm’s behavior under different conditions. As shown in Table 5, the performance increase on the HST dataset is more significant than that on the CWRU dataset. One main reason is that the CWRU dataset is much simpler than the HST dataset, and the model can achieve high accuracy without perturbation, which makes the improvement of the perturbation less significant. However, for the HST dataset, the model can benefit more from the perturbation, especially the sensor-wise perturbation, which can introduce more variations to the data and help the model learn more robust features.

The meticulous analysis reveals a notable trend: the addition of each type of perturbation led to an increase in accuracy. However, the most remarkable findings emerged when both data and model perturbations were simultaneously introduced, resulting in significantly enhanced accuracy compared to scenarios with either perturbation type alone, or none at all. This observation underscores the synergistic effect of combining diverse perturbation strategies, affirming their collective role in bolstering confidence reliability. Notably, the addition of any perturbation, whether data or model, consistently yielded improvements in accuracy, reaffirming the efficacy of perturbation injection in enhancing confidence reliability across various experimental setups.

**Figure 7:**
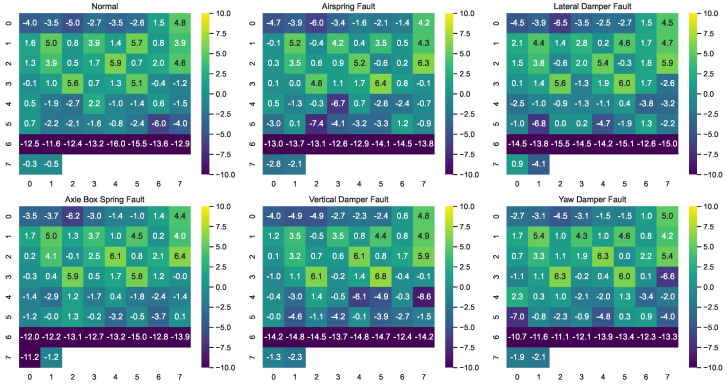
Visualization of the energy distribution of sensor-wise perturbations across different channels. The heatmap illustrates log-scaled energy values of injected perturbations.

To visually illustrate the impact of sensor-wise perturbation on different signal channels across various fault categories, a subset of samples from selected fault categories is chosen here to demonstrate the energy distribution of sensor-wise perturbation across different channels. For a clearer presentation, the log-scaled energy of injected perturbation is computed, followed by the generation of a heatmap for all 58 channels, as shown in Figure 7. From the figure, it can be observed that the energy distribution of perturbation varies across different channels, indicating the variability of sensor-wise perturbation in handling signals from different channels.

#### 3.2.3. Influence of Training Class Number

For few-shot methods, a high-way setting during model training is regarded as a viable approach to enhance model performance, as discussed in previous studies [12,15,23], wherein the model is trained on a higher number of classes than it is tested on. In the context of few-shot learning, a higher way setting introduces greater complexity by requiring the model to discriminate between a larger number of classes. The high-way setting during the training phase contributes to the generalization of the model’s ability to classify classes in the testing phase. Hence, in this section, experiments were conducted on the SPI-MCL method and TPN, two transductive few-shot learning methods, under different N-way training settings. It is worth noting that MAML, as a Model-Agnostic inductive few-shot learning method, maintains the same N-way settings in both training and testing phases; thus, experimentation with the MAML method is not undertaken here.

In Table 6, a comparison of the SPI-MCL and TPN methods’ performance on five-way and ten-way training settings is presented, examining their impact on the five-shot learning task performance. It can be observed that for the CWRU dataset, comprising only two channels and relatively easy to discern, the high-way training setting does not significantly affect performance. However, for the more challenging HST dataset, both TPN and our method exhibit noticeable performance improvements with the high-way training setting. Therefore, in practical applications, the adoption of high-way training settings is recommended to enhance the performance of fault diagnosis models, which can be feasible under certain conditions.

## 4. Conclusions and Future Work

This paper proposes a few-shot learning-based fault detection method for high-speed train suspension systems to address the challenge of limited fault samples in real-world scenarios. Leveraging few-shot learning principles and meta-confidence learning, the designed approach enhances the model’s robustness and generalization capability by incorporating sensor-wise perturbation. This perturbation method augments the main components of monitoring signals based on their characteristics, strengthening the model’s ability to learn sample distributions and generalize under limited data conditions. Experimental validation on both high-speed train fault datasets and publicly available benchmark bearing datasets, along with comparisons with other few-shot learning methods, demonstrate the effectiveness and superiority of the proposed approach. Furthermore, discussions and analyses on the effects of different perturbations and experiments on high-way settings during training provide guidance for practical applications. The proposed method achieves high accuracy in fault detection under limited sample conditions and is easily extendable to fault diagnosis problems in other domains.

Our future research includes exploring additional techniques to enhance the robustness of the proposed method under complex and dynamic operating conditions, as well as extending its applicability to diverse domains beyond high-speed train fault diagnosis. This may involve investigating advanced data augmentation strategies, exploring advanced information gain techniques in the presence of sensor-wise perturbations, and adapting the method to varying environmental conditions to ensure its effectiveness across a wide range of practical scenarios.

## Figures and Tables

**Table 1:** Split details for experiments on the high-speed train fault dataset.

Setting	Train Set	Test Set
Fault Locations *	Normal Lat (4 classes) Spr (8 classes) Yaw (8 classes)	Air (2 classes) Ver (8 classes)
Samples per class	500	500

* Note: Air → Air Spring, Lat → Lateral Damper, Yaw → Yaw Damper, Spr → Coil Spring, Ver → Vertical Damper.

**Table 2:** Split details for experiments on the CWRU Bearing Data.

Setting	Train Set	Test Set
Fault Locations *	Normal FE IR (3 classes) FE B (3 classes) FE OR centred (3 classes) FE OR orthogonal (2 classes) FE OR opposite (2 classes)	DE IR (3 classes) DE B (3 classes) DE OR centred (3 classes) DE OR orthogonal (2 classes) DE OR opposite (2 classes)
Samples per class	500	500

* Note: DE → drive end, FE → fan end, B → ball, IR → inner race, OR → outter race.

**Table 3:** The architecture of the backbone Resnet12 network.

Layer	Details
Convolution 1D	64 filters, 2 × 1 kernel, stride 1, padding 1
Max Pooling	2 × 2 kernel, stride 2
Residual Block ^†^ 1	3 × (64 filters, 3 × 1 kernel, stride 1, padding 1)
Residual Block ^†^ 2	3 × (128 filters, 3 × 1 kernel, stride 2, padding 1)
Residual Block ^†^ 3	3 × (256 filters, 3 × 1 kernel, stride 2, padding 1)
Residual Block ^†^ 4	3 × (512 filters, 3 × 1 kernel, stride 2, padding 1)
Pooling and Output *	-

* Note: The setting of the pooling and output layers is determined by the method used in the experiment. The prototype-based model does not output the class label directly, but the feature embedding. ^†^ Note: The residual block consists of two convolutional layers of one dimension with batch normalization and ReLU activation.

**Table 4:** Average detection performance over 1000 randomly generated episodes, with 95% confidence intervals. (The best results are highlighted in bold.)

Method	HST		CWRU	
	5 Way 1 Shot	5 Way 5 Shot	5 Way 1 Shot	5 Way 5 Shot
MAML [13]	$33.13{\%}_{\pm 3.90}$	$41.63{\%}_{\pm 10.89}$	$80.63{\%}_{\pm 2.12}$	$83.00{\%}_{\pm 1.41}$
TPN [15]	$26.34{\%}_{\pm 7.69}$	$51.14{\%}_{\pm 17.53}$	$67.37{\%}_{\pm 14.5}$	$76.51{\%}_{\pm 12.67}$
SPI-MCL-Instance	$84.54{\%}_{\pm 0.93}$	$93.93{\%}_{\pm 0.53}$	$98.60{\%}_{\pm 0.24}$	$98.58{\%}_{\pm 0.21}$
SPI-MCL-pair	$82.76{\%}_{\pm 0.90}$	$93.12{\%}_{\pm 0.53}$	$97.28{\%}_{\pm 0.37}$	$98.90{\%}_{\pm 0.17}$

**Table 5:** Average detection performance over 1000 randomly generated episodes under different perturbation settings, with 95% confidence intervals. (The best results are highlighted in bold.)

Method	HST		CWRU	
	5 Way 1 Shot	5 Way 5 Shot	5 Way 1 Shot	5 Way 5 Shot
SPI-MCL-Instance	$84.54{\%}_{\pm 0.93}$	$93.3{\%}_{\pm 0.53}$	$98.60{\%}_{\pm 0.24}$	$98.58{\%}_{\pm 0.21}$
SPI-MCL-Pair	$82.76{\%}_{\pm 0.90}$	$93.12{\%}_{\pm 0.53}$	$97.28{\%}_{\pm 0.37}$	$98.90{\%}_{\pm 0.17}$
SPI-MCL-Instance (NoSP)	$69.64{\%}_{\pm 1.16}$	$84.02{\%}_{\pm 0.96}$	$96.82{\%}_{\pm 0.41}$	$98.32{\%}_{\pm 0.23}$
SPI-MCL-Pair (NoSP)	$67.05{\%}_{\pm 1.22}$	$88.88{\%}_{\pm 0.78}$	$95.89{\%}_{\pm 0.49}$	$98.87{\%}_{\pm 0.18}$
SPI-MCL-Instance (NoMP)	$68.28{\%}_{\pm 1.26}$	$66.79{\%}_{\pm 1.20}$	$97.68{\%}_{\pm 0.31}$	$98.86{\%}_{\pm 0.17}$
SPI-MCL-Pair (NoMP)	$64.42{\%}_{\pm 1.20}$	$67.330{\%}_{\pm 1.25}$	$96.25{\%}_{\pm 0.46}$	$98.25{\%}_{\pm 0.23}$

Note: NoSP → without Sensor-wise Perturbation, NoMP → without Model Perturbation.

**Table 6:** Effect of training ways on 5-shot classification performance. (The best results are highlighted in bold.)

Setting	Method	5-Way 5-Shot	10-Way 5-Shot
HST	SPI-MCL-Instance	$93.93{\%}_{\pm 0.53}$	$99.65{\%}_{\pm 0.11}$
	SPI-MCL-Pair	$93.12{\%}_{\pm 0.53}$	$99.51{\%}_{\pm 0.09}$
	TPN [15]	$51.14{\%}_{\pm 17.53}$	$65.86{\%}_{\pm 17.30}$
CWRU	SPI-MCL-Instance	$98.60{\%}_{\pm 0.24}$	$98.53{\%}_{\pm 0.20}$
	SPI-MCL-Pair	$98.90{\%}_{\pm 0.17}$	$98.87{\%}_{\pm 0.16}$
	TPN [15]	$76.51{\%}_{\pm 12.67}$	$78.38{\%}_{\pm 12.97}$

## Data Availability

The data presented in this study are available on request from the corresponding author.
